# Silencing E3 Ubiqutin ligase ITCH as a potential therapy to enhance chemotherapy efficacy in p53 mutant neuroblastoma cells

**DOI:** 10.1038/s41598-020-57854-6

**Published:** 2020-01-23

**Authors:** Jinhong Meng, Aristides D. Tagalakis, Stephen L. Hart

**Affiliations:** 1 Ryboquin Ltd, Ettrick Riverside, Dunsdale Road, Selkirk, TD7 5EB UK; 2 Genetics and Genomic Medicine Programme, UCL Great Ormond Street Institute of Child Health, 30 Guilford Street, London, WC1N 1EH UK; 3 Present Address: Department of Biology, Edge Hill University, Ormskirk, L39 4QP UK

**Keywords:** Cancer therapy, Drug delivery

## Abstract

*P53* mutations are responsible for drug-resistance of tumour cells which impacts on the efficacy of treatment. Alternative tumour suppressor pathways need to be explored to treat *p53*- deficient tumours. The E3 ubiquitin ligase, ITCH, negatively regulates the tumour suppressor protein TP73, providing a therapeutic target to enhance the sensitivity of the tumour cells to the treatment. In the present study, two p53-mutant neuroblastoma cell lines were used as *in vitro* models. Using immunostaining, western blot and qPCR methods, we firstly identified that ITCH was expressed on p53-mutant neuroblastoma cell lines. Transfection of these cell lines with ITCH siRNA could effectively silence the ITCH expression, and result in the stabilization of TP73 protein, which mediated the apoptosis of the neuroblastoma cells upon irradiation treatment. Finally, *in vivo* delivery of the ITCH siRNA using nanoparticles to the neuroblastoma xenograft mouse model showed around 15–20% *ITCH* silencing 48 hours after transfection. Our data suggest that *ITCH* could be silenced both *in vitro* and *in vivo* using nanoparticles, and silencing of *ITCH* sensitizes the tumour cells to irradiation treatment. This strategy could be further explored to combine the chemotherapy/radiotherapy treatment to enhance the therapeutic effects on p53-deficient neuroblastoma.

## Introduction

Neuroblastoma is a childhood malignant tumour accounting for ~13% of all paediatric cancer mortality^1^. Some types of neuroblastoma are prone to developing drug-resistance during the course of treatment^2^. One of the genes responsible for this phenomenon is p53^3–6^, a well-characterized tumour suppressor protein, which binds to various transcriptional factors mediating apoptosis of tumour cells^7,8^. Mutations of *p53* lead to compromised tumour cell death and eventually the irresponsiveness to the chemotherapy treatment^6–8^.

Attempts to restore or reactivate the wide-type p53 function for tumour therapy^9–14^ are under investigation but it may be possible to utilize alternative pathways to compensate for the missing p53 function^15–17^. TP73 is a homologous molecule of p53 and shares significant sequence similarity particularly in the DNA binding domain (DBD), activation domain (AD) and tetramerization domain (TD)^18^. TP73 shows tumour suppressive activities through its ability to bind transcriptional target genes involved in apoptosis. Overexpression of wild type TP73 promotes the apoptosis of transformed cells. In addition, *p73* mutations are infrequent in human cancers^17^ including neuroblastomas^19,20^, making it an attractive gene to manipulate for therapeutic intervention of the p53-null tumours.

TP73 is expressed at low levels in normal tissues, but may be upregulated in some types of tumours^21–24^ or under conditions where p53 is inactivated^25^. The expression level of p73 protein is regulated by the E3 ubiquitin ligase ITCH^26^ *via* its ubiquitination pathway. Thus, inhibition of ITCH could elevate p73 expression and enhance the chemo-sensitivity of the tumour cells, especially those with defective p53^27^. In addition to p73, ITCH also regulates other tumour suppressor genes such as large tumour suppressor 1 (*LATS1*)^28,29^, *p63*^30,31^, and *RASSF5/NORE1*^32^, providing an attractive therapeutic target for tumour therapy. In this study, we tested the hypothesis that modulation of ITCH expression would enhance the sensitivity of the tumour cells to treatment. To do this, we used two TP53-null neuroblastoma cell lines as *in vitro* models, and used siRNA to downregulate ITCH expression. Furthermore, utilizing nanoparticles^33,34^, we tested the *in vivo* silencing efficacy of the candidate ITCH siRNAs in a neuroblastoma xenograft model.

Our study provides evidence that *ITCH* can be effectively silenced in neuroblastoma both *in vitro* and *in vivo*. Silencing of *ITCH* *in vitro* stabilizes TP73 protein on neuroblastoma cells and sensitizes the cells to irradiation treatment. Our results suggest that this novel strategy is feasible for combining with the conventional chemo-/radio-therapy to treat the drug-resistant TP53-null neuroblastomas.

## Results

### Expression of ITCH and TP73 in neuroblastoma cell lines

To determine the optimal *in vitro* cell culture model for this project, we chose two *p53* -mutant neuroblastoma cell lines, Kelly and BE2 cells, and performed semi-quantitative RT-PCR, real time qRT-PCR and immunostaining to determine the expression levels of *ITCH* and *p73* in these two cell lines.

**Figure 1:**
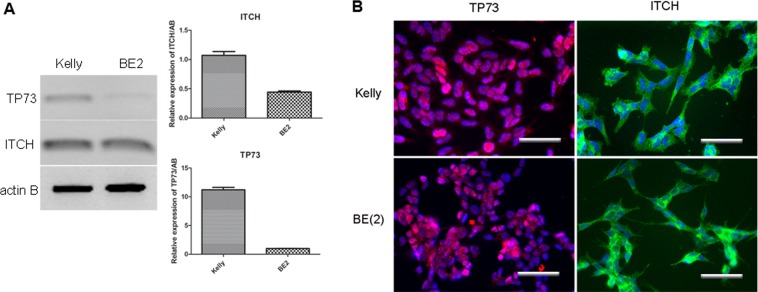
Expression of ITCH and TP73 in neuroblastoma cell lines. (**A**) RT-PCR and the qPCR results of the expression in Kelly cells and BE2 cells, (**B**) immunostaining showing the expression of ITCH and TP73 at the protein level, scale bar = 25 µm.

As shown in Fig. 1, RT-PCR on mRNA extracted from the proliferating Kelly cells and BE2 cells showed that both these two cell lines expressed *ITCH* and *TP73*. Real-time qRT-PCR suggested that Kelly cells express higher levels of *ITCH* and *TP73* than BE2 cells (Fig. 1A). Immunostaining showed that both cell lines also expressed ITCH and TP73 protein (Fig. 1B). Therefore, both cell lines could be used for transfections with ITCH siRNA in order to knockdown *ITCH* expression.

### Expression of integrin αv, β3 and β5 on neuroblastoma cells

It has been shown that nanoparticles containing peptide ME27, which contains an integrin-targeting RGD motif, can be an effective delivery tool for *in vivo* tumour targeting^35,36^ and we planned to use the same peptide for our *in vivo* silencing experiment. Thus, it was important to establish that the tumour cells expressed integrin receptor proteins to enable the specific targeting of the tumour by nanoparticles. Therefore, we examined the expression of the specific ME27 ligands, integrins αv, β3 and β5 in neuroblastoma cells by RT-PCR, immunostaining and western blot analysis.

**Figure 2:**
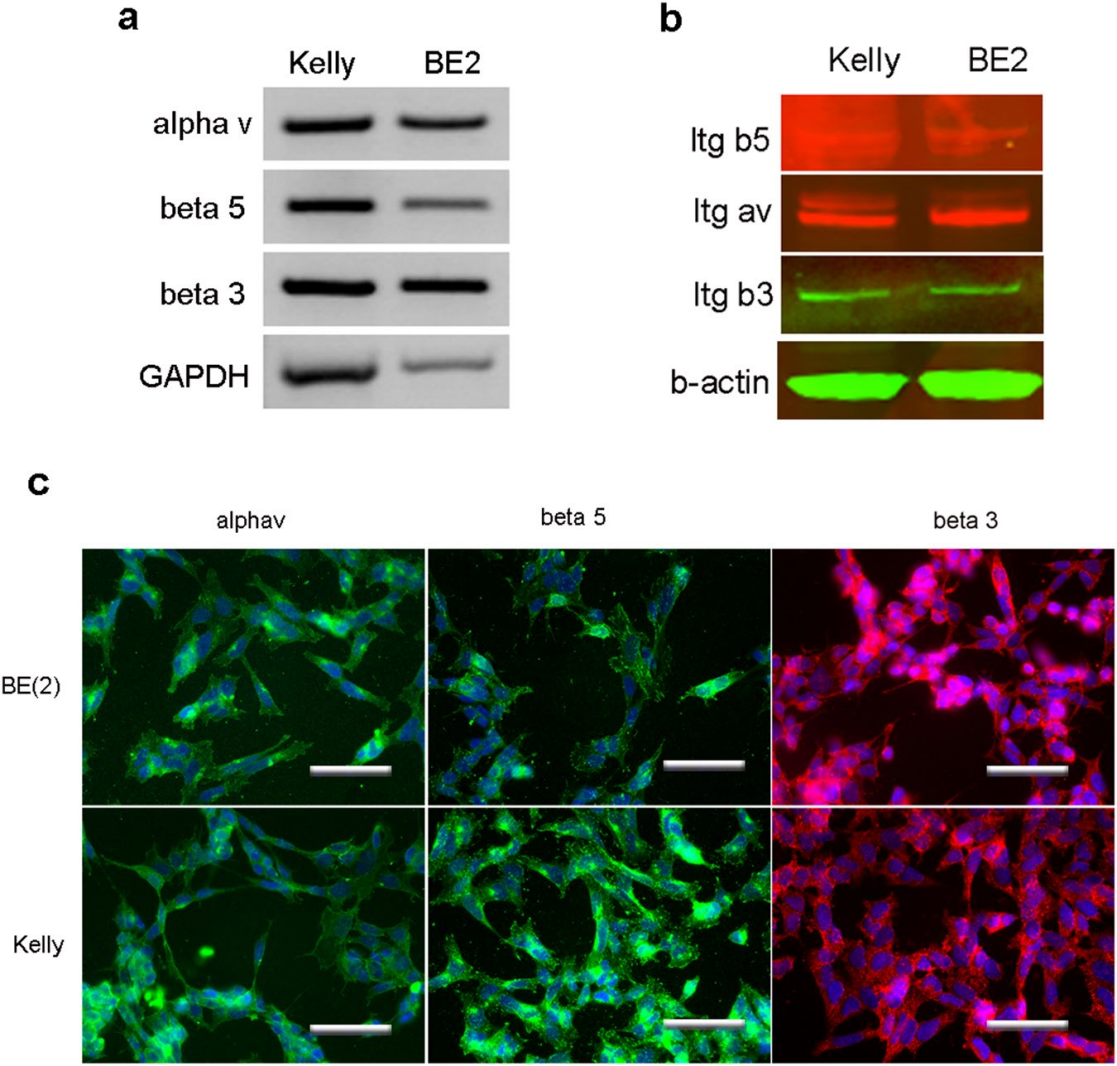
Expression of integrin αv, β3 and β5 in neuroblastoma cells. (**a**) RT-PCR; (**b**) western blot and (**c**) immunostaining all showed the presence of these integrin molecules in the neuroblastoma cell lines, Kelly and BE2, scale bar = 25 µm.

As shown in Fig. 2, we found that both Kelly and BE2 cells expressed integrins αv, β3 and β5 at the mRNA level (RT-PCR, Fig. 2a) and protein level (immunostaining, western blot, Fig. 2c,b). This result suggested that these neuroblastoma cells can be targeted by the nanoparticles via the interaction between the ME27 peptide and integrins.

### *In vitro* silencing of ITCH in neuroblastoma cell lines

#### Transfection using Lipofectamine 2000 (L2K) reagent

To determine *ITCH* silencing in neuroblastoma cells *in vitro*, we firstly transfected neuroblastoma cells with ITCH siRNA, using L2K as the transfection reagent. To determine the optimal condition of the siRNA transfection, cells were transfected with either a) ITCH siRNA (6.25 nM to 100 nM) using 2 µl/well L2K, or b) Different amounts of L2K (0.5 µl/well, 1 µl/well and 2 µl/well in 24-well plate) with 100 nM ITCH siRNA for 4 hours in OptiMEM, and then change into complete medium for a further 72 hours. The ITCH mRNA levels were determined by qRT-PCR, and cells transfected with irrelevant siRNA were used as the control.

#### Effects of *s*iRNA concentration on ITCH mRNA knockdown in neuroblastoma cells

**Figure 3:**
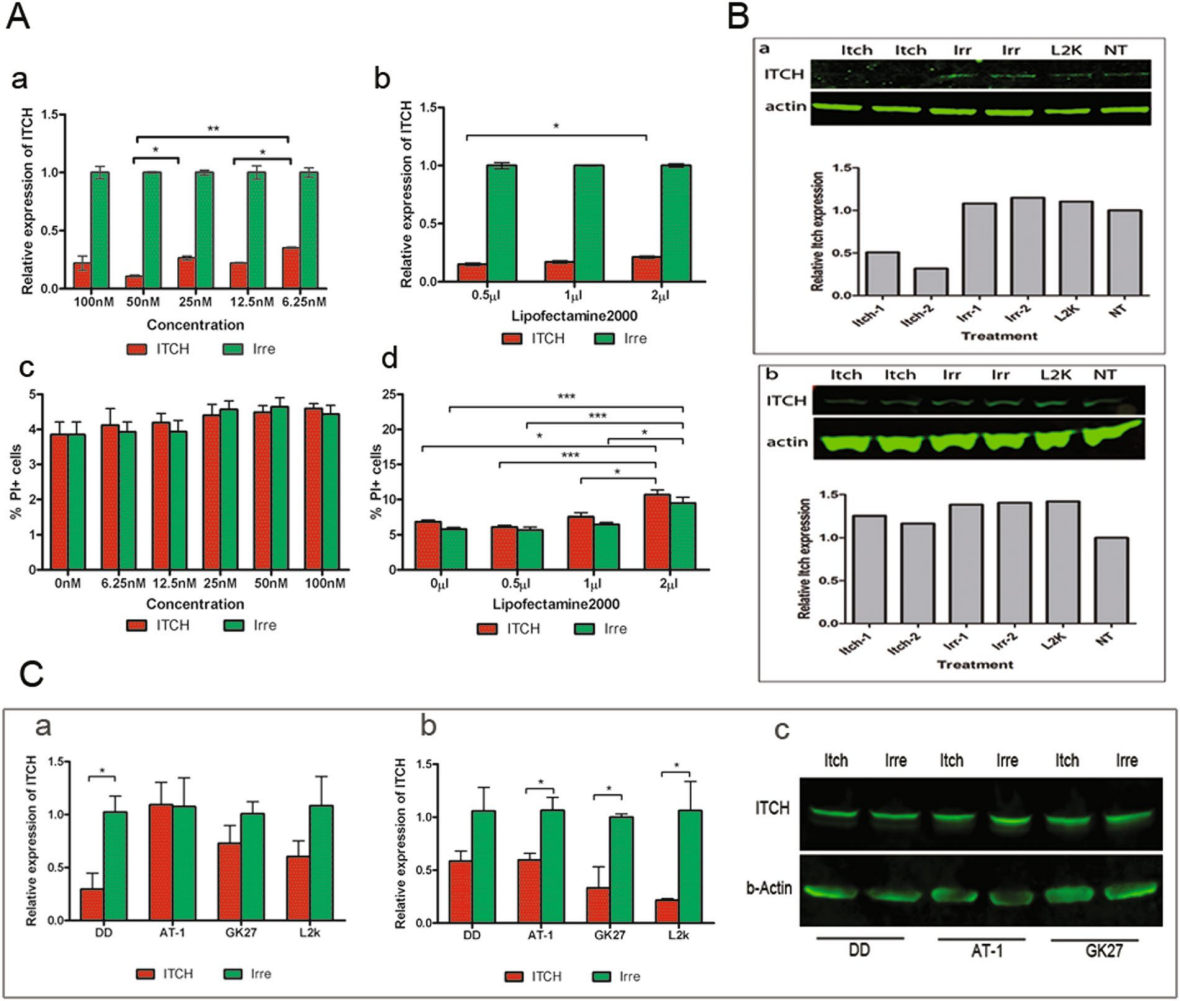
Knockdown of ITCH *in vitro* using LipofectAMINE2000 (L2K) (**A**,**B**) or nanoparticles (**C**). (**A**) qPCR of ITCH mRNA (a and b) of Kelly cells transfected with different concentrations of ITCH siRNA (a) or using different amounts of L2K reagent (b). The cell viability in each transfection condition is indicated by %PI + cells after transfection (c,d). (**B**) Western blot showed the knockdown of ITCH protein by siRNA transfection in Kelly cells (B-a) or BE2 cells (B-b). L2K = L2K only. (**C**) Expression of ITCH mRNA (C-a and C-b) and ITCH protein (C-c) on Kelly cells after transfection of 100 nM ITCH siRNA with different nanoparticle formulations for 4 hours (C-a) or 48 hours (C-b). ITCH protein level is measured by western blot 48 hours after transfection (C-c).

In Kelly cells, after transfection, the level of ITCH mRNA was 22 ± 3.6%, 11 ± 6%, 26.5 ± 1%, 22.2 ± 0.3% and 35.3 ± 0.3% in 100 nM, 50 nM, 25 nM, 12.5 nM and 6.25 nM ITCH siRNA transfected groups, respectively, of that transfected with corresponding irrelevant siRNAs (Fig. 3A). Kruskal-Wallis test followed by multiple comparisons suggested that there were significant dose-dependent differences between cells transfected with 50 nM and 25 nM, or 50 nM and 6.25 nM, or 12.5 nM and 6.25 nM ITCH siRNAs. Cells transfected with 50 nM ITCH siRNA showed the best knockdown efficiency. Similarly, in BE2 cells, there were 63.8 ± 7.8%, 26.5 ± 1.8%, 50.3 ± 1.7%, 44.6 ± 2.1% and 134.5 ± 0.4% ITCH mRNA expression in 100 nM, 50 nM, 25 nM, 12.5 nM and 6.25 nM ITCH siRNA transfected groups, respectively, in comparison to that transfected with corresponding irrelevant siRNAs. Kruskal-Wallis test followed by multiple comparisons suggested there were dose- dependent differences between 50 nM and 6.25 nM ITCH siRNA transfected groups (Supplementary Fig. 1). Cells transfected with 50 nM ITCH siRNA showed the best knockdown efficiency.

To determine the potential dose-related toxicity of siRNA, we examined the cell viability after transfection of different concentrations of siRNA. FACS analysis of PI staining showed that there was no dose-dependent toxicity on Kelly cells (Fig. 3A); while 100 nM siRNA-transfected BE2 cells contained significant more PI + cells than non-transfected cells and 6.25 nM siRNA-transfected cells, evidence of toxicity of high dose siRNA on BE2 cells (Supplementary Fig. 1).

#### Effects of amount of L2K on ITCH mRNA knockdown in neuroblastoma cells

In Kelly cells, using 100 nM siRNA, 15 ± 0.6%, 17 ± 0.6% and 21.2 ± 0.6% ITCH mRNA were detected in cells transfected with 0.5 µl/well, 1 µl/well and 2 µl/well L2K, respectively, in comparison to corresponding irrelevant siRNA transfected cells (Fig. 3A). In BE2 cells, 4.2 ± 0.7%, 48.4 ± 0.6% and 71.9 ± 0.6% ITCH mRNA was observed in cells transfected with 0.5 µl/well, 1 µl/well and 2 µl/well L2K, respectively, in comparison to corresponding irrelevant siRNA-transfected cells (Supplementary Fig. 1). Cell viability analysis on both cell lines showed that both 0.5 µl/well and 1 µl/well transfected cells contained similar amount of PI + cells to that of control cells, while 2 µl/well transfected cells contained significant more PI + cells than non-transfected cells (Fig. 3A and Supplementary Fig. 1). These results suggested that the cells transfected with lower amounts of transfection reagent (0.5 µl/well and 1 µl/well) have higher transfection efficiency, with lower toxicity than cells transfected with a higher amount of reagent (2 µl/well).

#### Knockdown of ITCH protein in neuroblastoma cells using L2K reagent

Next, we tested the extent of ITCH silencing at the protein level in neuroblastoma cells, using L2K as transfection reagent. Kelly and BE2 cells were transfected with 50 nM ITCH siRNA/irrelevant siRNA and 0.5 µl/well L2K, and cell lysates were collected 72 hours after transfection. As shown in Fig. 3B, the ITCH protein level was 31.6% in ITCH siRNA-transfected Kelly cells (Fig. 3B-a), in comparison to non-transfected cells. However, there were no changes in ITCH protein level in treated BE2 cells in comparison to irrelevant siRNA-treated cells (Fig. 3B-b).

Based on the western blot results from the Kelly and BE2 cells, we determined that the Kelly cell line was more responsive to the ITCH siRNA treatment than the BE2 cell line, in terms of silencing at the protein level. Therefore, in the *in vivo* experiment that followed, we chose to use Kelly cells to establish the xenograft models.

### Transfection using nanoparticles

We also transfected Kelly cells with nanoparticles containing liposomes formulated with different concentration of polyethylene glycol (PEG)^35^. Three types of lipids were used as following: DOTMA/DOPE, with no PEG; AT-1, containing 1% PEG and GK27, which contains 5% PEG. Kelly cells were plated in 24-well plates at a density of 8 × 10^4^ cells/well. On the day of the transfection, cells were transfected with the following formulations: 1) DOTMA/DOPE: ME27: ITCH siRNA; 2) AT-1:ME27: ITCH siRNA and 3) GK27:ME27: ITCH siRNA, all at a ratio of 1:4:1. Cells transfected with irrelevant siRNA with corresponding formulations were used as negative controls. Cells transfected with L2K and ITCH siRNA were also included as a positive control. The concentration of siRNA used in this experiment was 100 nM. All transfections were performed in OptiMEM and 2 time points were compared in this experiment: 4 hours (nanocomplexes left for 4 hours on cells and then replaced with complete media) and 48 hours after transfection (nanocomplexes left for 48 hours on cells in complete media).

As shown in Fig. 3C, when the cells were transfected for 4 hours, there was 70% knockdown (29.6 ± 15% ITCH mRNA) of ITCH in the DOTMA/DOPE:ME27:ITCH siRNA-transfected group. However, AT-1:ME27:ITCH siRNA and GK27:ME27:ITCH siRNA-transfected groups achieved either none (109 ± 21% ITCH mRNA) or small amount (around 22% knockdown, 72.8 ± 16.9% ITCH mRNA) of ITCH silencing (Fig. 3C-a). However, when the cells were transfected for 48 hours (Fig. 3C-b), which means that the nanoparticles stay in the medium throughout the experiment, there was around 42% knockdown (58.4 ± 9.5% of ITCH mRNA) of ITCH in DOTMA/DOPE:ME27:ITCH siRNA-transfected group, 37% knockdown (59.5 ± 6.3% ITCH mRNA) in AT-1:ME27:ITCH siRNA-transfected group, and around 61% knockdown (33 ± 20% ITCH mRNA) of ITCH in GK27:ME27:ITCH siRNA-transfected group.

These data suggested that for the DOTMA/DOPE:ME27:ITCH siRNA formulation, extending the incubation time did not enhance the transfection efficiency; while for AT-1:ME27:ITCH siRNA and GK27:ME27:ITCH siRNA formulations, increasing the transfection time significantly enhanced the transfection efficiency.

Next we examined the knockdown of ITCH protein after nanoparticle transfection. Kelly cells were transfected with DOTMA/DOPE:ME27:ITCH siRNA, AT-1:ME27:ITCH siRNA and GK27:ME27:ITCH siRNA for 48 hours; cells transfected with irrelevant siRNA were used as control. Unlike the cells transfected using L2K reagent, cells transfected using nanoparticles showed no difference in ITCH protein level in ITCH siRNA transfected samples and the irrelevant siRNA transfected samples, as indicated by western blot analysis (Fig. 3C-c).

### Knockdown of ITCH upregulates TP73 in Kelly cells

**Figure 4:**
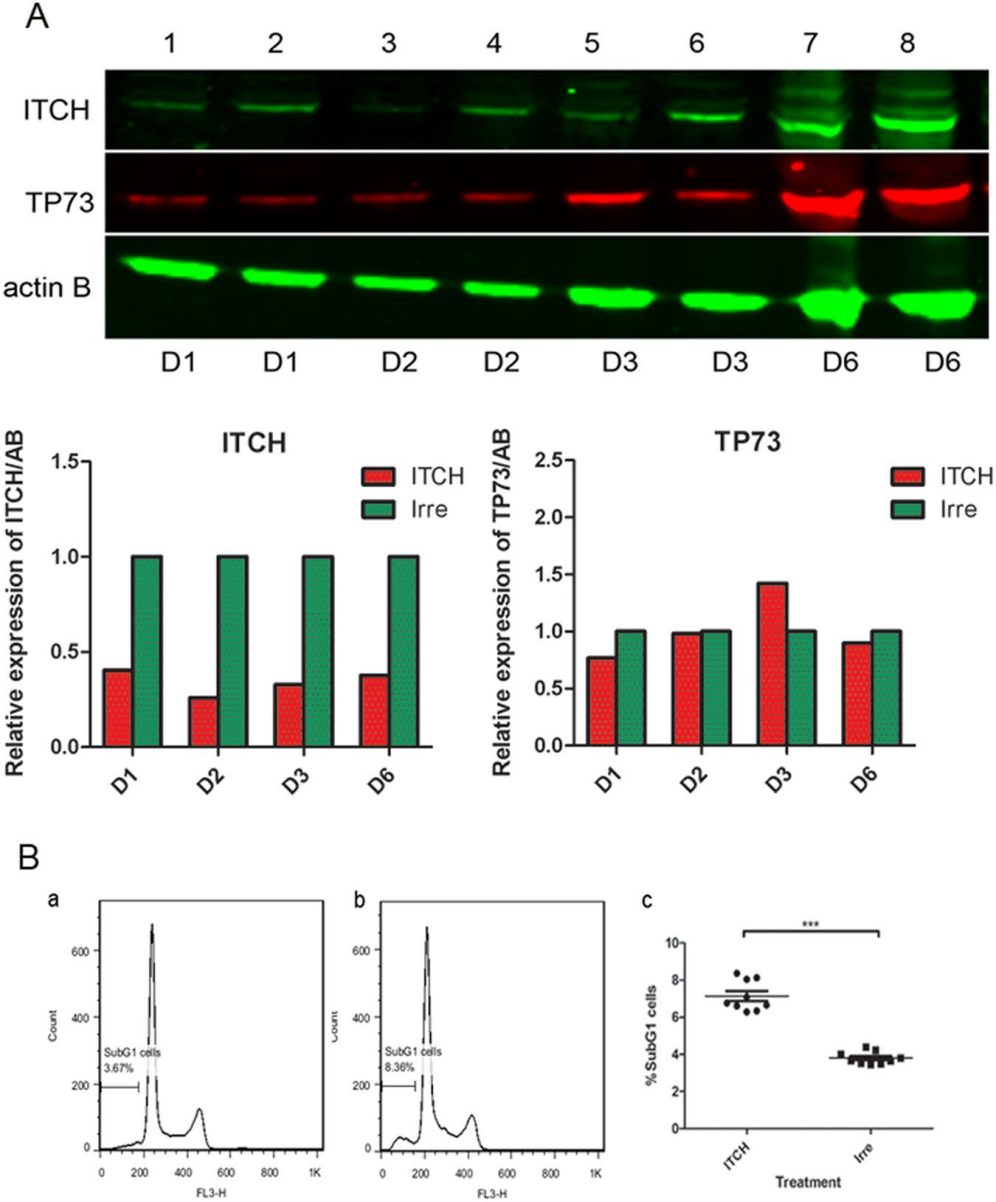
Knockdown of ITCH causes the upregulation of TP73 (**A**) and induces apoptosis of Kelly cells upon irradiation. (**A**) Western blot showing silencing of ITCH protein and upregulation of TP73 after transfection of ITCH siRNA in Kelly cells. 1, 3, 5 and 7 are samples transfected with ITCH siRNA; 2, 4, 6 and 8 are samples transfected with irrelevant siRNA. D1, D2, D3 and D6 suggested days after transfection. (**B**) Apoptotic cells after irradiation of Kelly cells which have been transfected with ITCH siRNA (b) and irrelevant siRNA (a). Quantification of percentage of SubG1 cells within each treatment group (c).

As TP73 is the major protein targeted by ITCH, we next examined the level of TP73 protein in ITCH siRNA-transfected cells. Kelly cells were transfected with ITCH siRNA or irrelevant siRNA in 6-well plates using  L2K. The protein samples were collected 1 day (D1), 2 days (D2), 3 days (D3) and 6 days (D6) after transfection. Western blot analysis with the ITCH and TP73 antibodies was performed using the above samples, and the expression of each protein was normalized to β-actin (Fig. 4).

As expected, downregulation of ITCH protein was observed in samples which had been transfected with ITCH siRNA, in comparison to samples which were transfected with irrelevant siRNA, from 1 day to 6 days after transfection. However, we only detected TP73 upregulation at day 3 samples. These data suggest that downregulation of ITCH by siRNA transfection could subsequently induce the stabilization of TP73 in treated cells, after the ITCH protein has been downregulated. What is more, TP73 upregulation is transient, which could not be detected at 6 days after the ITCH siRNA transfection.

### Knockdown of ITCH sensitizes Kelly cells to irradiation

Our hypothesis is that upon silencing of ITCH, the tumour suppressor protein TP73 would be stabilized which, theoretically for p53 mutant cells, would trigger the apoptotic pathway upon the drug/irradiation treatment. Kelly cells were transfected with ITCH and irrelevant siRNA, respectively. Three days later, cells were subjected to 4 Gy irradiation. The subG1 population was analyzed one day after the irradiation. As shown in Fig. 4B, one day after irradiation, cells which have been transfected with ITCH siRNA contained 8.36% apoptotic cells, while cells which have been transfected with irrelevant siRNA contained 3.56% SubG1 cells. This difference was statistically significant between the 2 groups (p < 0.001, Mann-Whitney test).

In conclusion, we have shown that silencing of ITCH by siRNA significantly induced apoptosis of Kelly neuroblastoma cells upon irradiation.

### *In vivo* silencing of ITCH in neuroblastoma xenografts

#### The relative expression of ITCH in the xenograft tumours was decreased at 48 hours after transfection, not at 24 h

**Figure 5:**
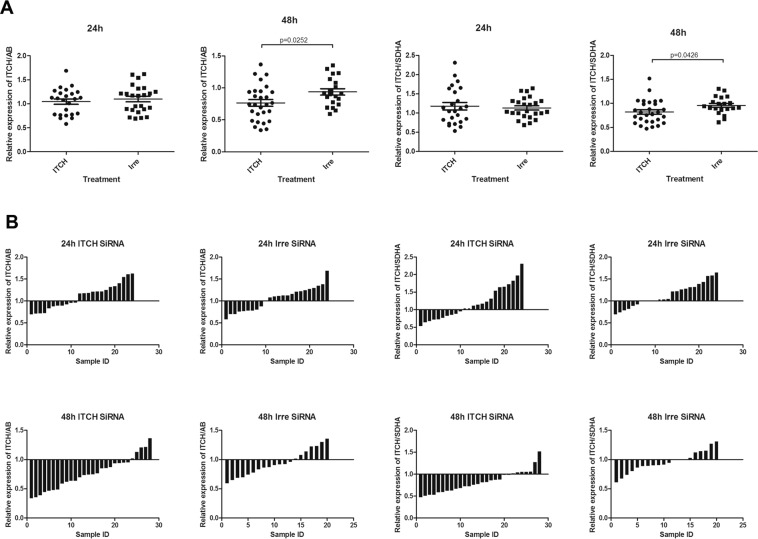
ITCH expression in tumours 24 hours and 48 hours after ITCH siRNA treatment. (**A**) Relative ITCH expression in each treatment group. (**B**) Individual sample responses to the ITCH siRNA treatment at 24 hours or 48 hours. There were no differences between the treatment group and the control group 24 hours after treatment. There were, however, more samples with lower levels of ITCH expression in the treated group than the control group 48 hours after treatment.

As shown in Table 1, the relative expression of ITCH at 24 hours in the ITCH siRNA- treated group was similar to that in the irrelevant siRNA- treated group, when the data were normalized to either β-actin or SDHA^37^, there were no statistical differences between these 2 groups (Fig. 5A) by either normalization method. However, 48 hours after delivery, we found that the relative expression of ITCH was significantly lower in the treated group than in the control group (Fig. 5A), when the data were normalized to either β-actin (p = 0.0252, 19.15% silencing) or SDHA (p = 0.0426, 14.6% silencing).

**Table 1:** The relative expression of ITCH in the xenograft tumours.

	Relative expression of ITCH/β-actin (mean ± SEM)		Relative expression of ITCH/SDHA (mean ± SEM)	
	24 hours	48 hours	24 hours	48 hours
ITCH siRNA	1.05 ± 0.06	0.76 ± 0.05	1.18 ± 0.10	0.82 ± 0.05
Irrelevant siRNA	1.10 ± 0.06	0.94 ± 0.05	1.13 ± 0.06	0.96 ± 0.04
P value	p > 0.05	p = 0.0252	p > 0.05	p = 0.0426

#### Individual sample response to ITCH siRNA treatment showed that more samples in the treated group 48 hours after treatment express lower levels of ITCH mRNA

We then plotted all the individual samples for their relative expression of ITCH, in comparison to control sample. Fig. 5B shows that at 24 hours after transfection there were no differences in ITCH expression between the ITCH siRNA treated group and irrelevant siRNA treated group. However, the treated group at 48 hours after treatment showed significantly decreased levels of ITCH expression than the control group. The tendency was the same when the samples were normalized with either β-actin or SDHA (Fig. 5B).

**Figure 6:**
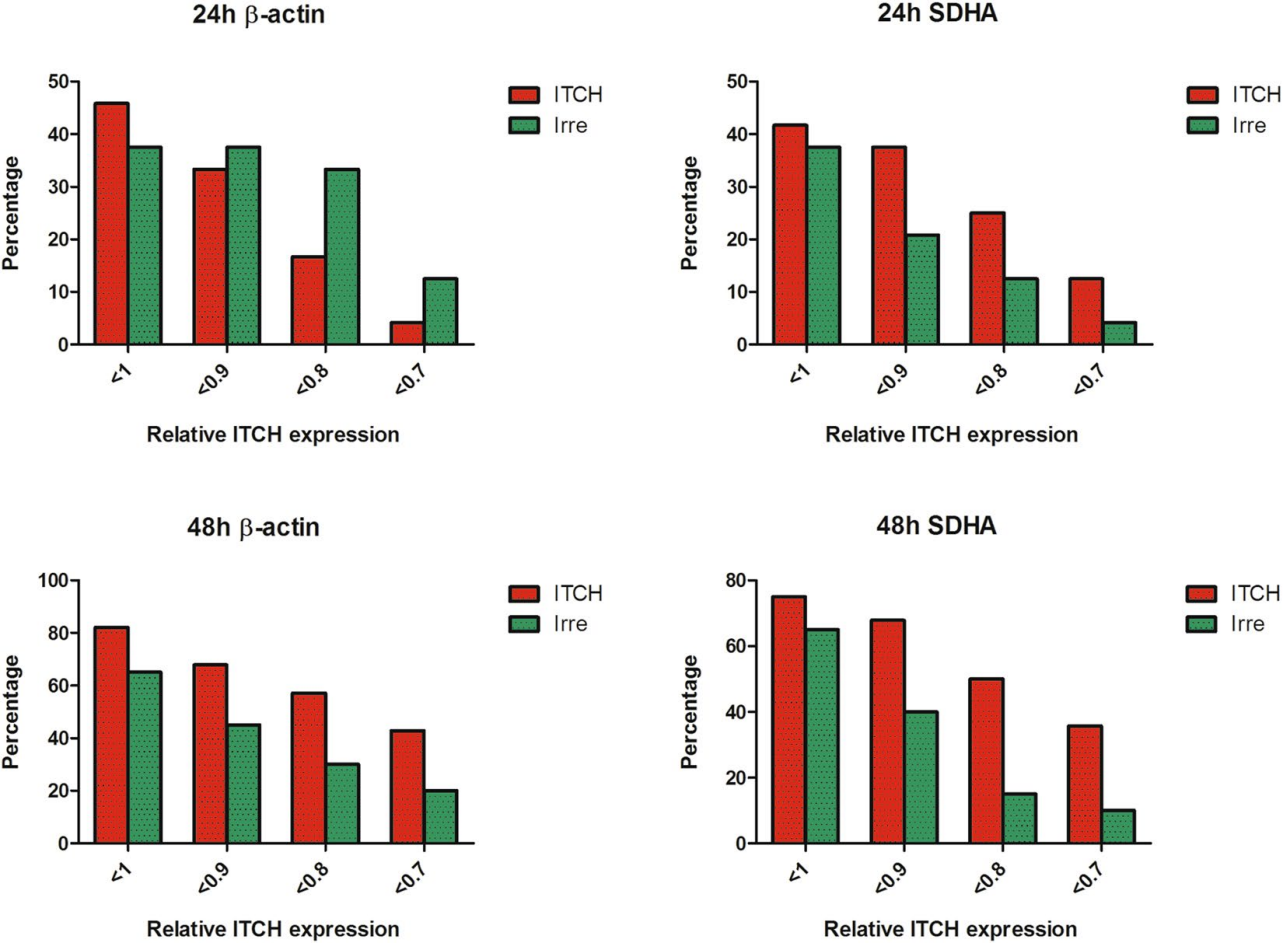
Percentage of tumour samples of each treatment group which express ITCH mRNA relatively lower than 1, 0.9, 0.8 and 0.7, respectively.

We also compared the percentage of low-ITCH expression samples in each group. The percentage of low-ITCH expression samples (relative expression lower than 1 or 0.8) within each group was calculated, and the differences between treated and control group is shown in Fig. 6. We found that 24 hours after delivery, there were only slightly more or even less samples expressing lower levels of ITCH in the treated group, while at 48 hours after delivery, there were 27.14% or 35% more samples expressing lower levels of ITCH (relative expression <0.8) in the treated group than in the control group, when the data were normalized to β-actin or SDHA, respectively.

## Discussion

Current therapy for neuroblastoma, involves surgery, chemotherapy, monoclonal antibody treatment, and radiotherapy^38^ with efficacy of each treatment depending on the disease stage and its biological features^39^. Surgery alone may achieve remission of MYCN-amplified neuroblastoma if it remains localized^38^ but usually patients receive 13-cis-retinotic acid treatment to induce neuronal differentiation^40^ and maintain a minimal residue disease^41^ although patients frequently develop 13-cis-retinotic acid resistance leading to relapse^42^. Anti-GD2 antibody therapies combined with granulocyte macrophage colony stimulating factor (GM-CSF), interleukin-2 (IL-2) and 13-cis-retinotic acid have improved the 2-year survival rate by 20%^40,43,44^, but neuroblastoma cells with low GD2 expression fail to respond to this therapy^45^ and so the prognosis of high-risk neuroblastoma remains very poor^43,46^ and new treatments are required. We have previously described nanoformulations for delivery of plasmid DNA encoding IL-2 and IL-12^35,36^ for therapy of murine form of neuroblastoma but here we explore a new approach in human p53-deficient neuroblastoma cells based on siRNA delivery to silence ITCH ligase and so induce p73 expression to mediate apoptosis in response to DNA damaging agents or radiation. Although this approach has been described elsewhere for therapy of pancreatic cancer^33^, we have explored its potential for the first time in developing a novel therapy for neuroblastoma.

Mutations of the tumour suppressor gene* p53* are the major reason for recurrent, drug-resistant neuroblastoma^5^. In addition to restoring the functional p53 pathway, manipulation of alternative tumour suppressor pathways may provide therapeutic benefit for treatment of these types of tumours. In this study, we aimed to explore the possibility of elevating the TP73 protein, a homologous protein of the p53 superfamily, by inhibiting its ubiquitinylation pathway, to elicit the apoptosis of p53-mutant neuroblastoma cells. The molecule we chose to target is ITCH, the E3 ubiquitin ligase responsible for the degradation of a panel of tumour suppressor genes, including TP73. In this study, we showed that ITCH and TP73 were expressed in two p53 mutant neuroblastoma cell lines, with Kelly cells expressing higher levels of both ITCH and TP73 than BE2 cells (Fig. 1), suggesting the existence of a compensating tumour suppressor pathway in the absence of functional p53 in these cell lines, and the feasibility of manipulating this pathway to induce the programmed cell death of tumour cells. ITCH, an E3 ubiquitin ligase, is the key enzyme responsible for ubiquitinylation and degradation of the TP73 protein^26^. Therefore, downregulation of ITCH expression in tumour cells undoubtedly provides therapeutic value for treatment of the p53-mutant tumours. In our *in vitro* transfection experiment, although both cell types could be transfected and achieved silencing of ITCH at the mRNA level, BE2 cells failed to respond at the protein level (Fig. 3B). Therefore, we focused on Kelly cells as the *in vitro* and *in vivo* model in the following studies.

The *p73* gene has two distinct promoters coding for two protein isoforms with opposite effects: the transactivation-proficient TAp73 shows pro-apoptotic effects and the amino-terminal-deleted DeltaNp73 has an anti-apoptotic function. The cellular outcome of the tumour cells upon treatment is finely regulated by the balance between TAp73 and DeltaNp73 in the *p53*-mutant cells^15^. It has been shown that the major isoform of TP73 expressed in Kelly cells was the TAp73^47^, whose upregulation would enhance the apoptosis of the cells upon treatment. Our western blot using a p73 antibody also confirmed the single TAp73 band at a molecular weight of around 73 KD (Fig. 4A). After ITCH silencing, the TP73 was upregulated at the protein level in Kelly cells and consequently these cells which were initially unresponsive, underwent apoptosis upon irradiation, as indicated by an increased SubG1 population (Fig. 4B). We found that downregulation of ITCH protein was observed in samples which had been transfected with ITCH siRNA, in comparison to samples which were transfected with irrelevant siRNA, from 1 day to 6 days after transfection. However, we only detected TP73 upregulation in the sample transfected 3 days before (Fig. 4A). This is expected as in the biological process, the TP73 protein level is regulated by the E3 ubiquitin ligase ITCH: in the presence of ITCH protein, TP73 is polyubiquitinated, targeting it for degradation. Once the ITCH protein is downregulated by siRNA silencing, the ubiquitination of TP73 is also inhibited, leading to accumulation of the TP73 in the cells. However, as these are sequential biology processes, it is understandable that the decrease of ITCH and the increase of TP73 do not happen at the same time point; but rather occur in a stepwise manner.

We also noticed that the extent of TP73 protein upregulation was not as dramatic as that of the ITCH silencing, and the increase of the SubG1 cell population was only mild in ITCH siRNA-transfected Kelly cells compared to irrelevant siRNA-treated cells. This suggested that other TP73-independent mechanisms may also be involved in ITCH-mediated tumor cell apoptosis, because TP73 is not the sole protein regulated by ITCH. Other molecules such as TP63^30^, large tumour suppressor 1 (LATS1)^28,29,48^, and RASSF5/NORE1^32^ have also been reported to be negatively regulated by the ITCH protein.

In our experiments, we used nanoparticles as a carrier system to deliver the ITCH siRNA *in vivo*. This was based on our previous findings that the receptor-targeted liposome-peptide-siRNA nanoparticle is an efficient delivery system for *ex vivo* and *in vivo* applications^49–53^. The formulation of the nanoparticles contains peptide ME27 which can mediate receptor-specific targeting *via* its interaction with integrin αvβ3 and αvβ5 on cells. Integrin αvβ3 is a well-characterized cell adhesion molecule which is highly expressed on active angiogenic endothelium and glioblastoma tumor cells^54^, and being used as a targeting molecule for radioimmunotherapy of glioblastoma *in vivo*. We found that these integrins were present in neuroblastoma cell lines (Fig. 2), suggesting that nanoparticles formulated with our experimental design could effectively target tumour sites *via* the receptor-peptide interaction after *in vivo* delivery into a neuroblastoma xenograft model. In addition to determining the expression of integrins in neuroblastoma cell lines, we performed *in vitro* transfections with nanoparticles where we compared the transfection efficiency of the lipid formulations which contained 1% PEG (AT-1), 5% PEG (GK27) and no PEG (DOTMA/DOPE). The presence of PEG has been shown to stabilize the nanoparticles in serum *in vivo*, preventing aggregation or disassembly^50^. We found that the lipid formulations containing PEG (AT-1 and GK27) did not work as well as the lipid without PEG (DOTMA/DOPE) in 4 hour transfections (Fig. 3C), although at longer incubation times (48 hours), ITCH siRNA silencing was evident, suggesting a slow-releasing process with these nanoparticles in cell culture. This was expected as it is well known that PEGylation, although improving nanoparticle stability in the presence of serum proteins, can also reduce transfection efficiency by retarding cell membrane interactions^55^.

Indeed, our *in vivo* results showed a similar tendency of slow release of siRNA from the nanoparticle formulations. 24 hours after transfection, there was no silencing detected in the treated groups, while 48 hours after transfection silencing was detectable by qPCR, with statistically lower levels of ITCH expression in the ITCH siRNA-treated group than in the irrelevant siRNA-treated group (Fig. 5A). There were also more samples expressing lower ITCH mRNA levels in the treated group than in the control group 48 hours after transfection (Fig. 5B). This result was consistent with our *in vitro* findings and therefore, a combination of ITCH siRNA and the chemo/radiotherapy on xenograft models will be performed in the future to further evaluate the therapeutic effect of manipulating ITCH expression in the treatment of drug-resistance neuroblastoma.

This study shows for the first time the potential for a siRNA therapy in treating neuroblastoma which would potentially be used to enhance the efficacy of radiation therapy or other chemotherapeutic drugs designed to induce apoptosis. The strategy of augmenting the efficacy of conventional cancer therapies with siRNA could be very effective and clinically attractive.

## Methods

### Ethics approval

All *in vivo* procedures were approved by UCL animal care policies and were carried out under Home Office Licenses issued in accordance with the United Kingdom Animals (Scientific Procedures) Act 1986 (UK).

### Materials

1,2-di-O-octadecenyl-3-trimethylammonium propane (DOTMA), 1,2-dipalmitoyl-*sn-*glycero-3-phosphoethanolamine-N-[methoxy(polyethylene glycol)-2000] (DPPE-PEG2000) and 1,2-dioleoyl-sn-glycero-3-phosphoethanolamine (DOPE) were purchased from Avanti Polar Lipids, Inc. (Alabaster, AL, USA). Peptide ME27 (K16RVRRGACRGDCLG) was synthesized by Alta Bioscience (Birmingham, UK).

### Cell culture

Two p53-mutant neuroblastoma cell lines, Kelly^47,56^ and SK-N-BE-2 (BE2) cells^5^ were maintained *in vitro* in RPMI-1640 medium (Sigma, Dorset, UK) supplemented with 2 mM Glutamine and 10% FBS (Thermo Fisher, Paisley, UK). Cells were split every 3–4 days.

### Transfection with Lipofectamine 2000 (L2K)

One day before transfection, cells were plated at 8 × 10^4^ cells per well for 24-well plates or 1.5 × 10^5^ cells per well for 6-well plates in culture medium. On the day of transfection, L2K (Thermo Fisher, Paisley, UK) and ITCH/Irrelevant siRNA (Eurogentec, Southampton, UK) were pre-diluted in OptiMEM (Thermo Fisher, Paisley, UK) at various concentrations and mixed to form complexes at room temperature (RT) for 25 min before adding to cells. mRNAs or protein lysates were collected 72 hours after transfection for further analysis. For cell viability assay, transfected cells were collected 72 hours after transfection and stained with 1 µg/ml Propidium Iodide (PI). Percentage of PI + cells in each group was determined using a FACS calibur machine (BD, Wokingham, UK). Data were analyzed using the Flowjo software (Flowjo, LLC).

### Transfection with nanoparticles *in vitro*

Nanoparticles were formulated as a mixture of Lipid:peptide:siRNA, as described previously^52,57^. In this study, we pre-formulated 3 different liposomes with different concentrations of PEG; 1) DOTMA/DOPE (DD), containing no PEG; 2) AT-1, DD containing 1% PEG; and 3) GK27, DD which contained 5% PEG. The peptide used in this study is ME27. The siRNA used is ITCH siRNA (Eurogentec, Southampton, UK, sense strand: 5′ GCU-GUU-GUU-UGC- CAU-AGA-A55 3′; antisense strand: 5′ UUC-UAU-GGC-AAA- CAA-CAG-C55 3′) and Irrelevant siRNA (Dharmacon, UK, D-001810, on-target plus Non-targeting pool). The nanocomplexes were made at a Lipid:Peptide:siRNA weight ratio of 1:4:1, and the siRNA concentration used for transfection was 100 nM. Kelly cells were seeded at 8 × 10^4^ cells/well in 24-well plates one day before transfection. Nanoparticles were carefully prepared and mixed in the order of Lipid + Peptide + siRNA, and left at RT for 30 min in order to form complexes before adding to the cells. The whole plate was centrifuged at 1500 rpm for 5 min to allow the nanoparticles to settle and incubated for either 4 hours or 48 hours before analysis.

### Semi-quantitative RT-PCR

RNA of cultured cells was extracted using RNeasy mini kit (Qiagen, Manchester, UK). RNA concentration was determined by NanoDrop 3.1 software. A one-step RT-PCR kit (Qiagen, Manchester, UK) was used to perform the semi-quantitative RT-PCR, using 10 ng RNA from each sample. The sequences of the primers are: ITCH forward: 5′-ACCGGCTGCCATCTTAGTCT-3′, ITCH reverse: 5′-GGAAAACCTGAAGTTCTCACAGT-3′; beta-actin forward: 5′-GCCCTGAGGCACTCTTCCA-3′, beta-actin reverse: 5′-ATGCCACAGGACTCCATGC-3′; SDHA forward: 5′-TGGGAACAAGAGGGCATCTG-3′, SDHA reverse: 5′-CCACCACTGCATCAAATTCATG-3′.

### Real-time PCR

cDNA from each RNA sample was synthesized using superscript III first strand synthesis kit (Thermo Fisher, Paisley, UK). To determine the ITCH expression, real time PCR was performed using SYBR-green qPCR kit (Eurogentec, Southampton, UK), using beta-actin or SDHA (sequence details as above) as endogenous control. The qPCR was run using either StepOne plus qPCR machine (Thermo Fisher, Paisley, UK) or CFX96 qPCR system (Bio-Rad, Watford, UK). Results were analysed using their corresponding software.

### Immunostaining on cultured cells

Cells were plated on poly-lysine coated coverslips (VWR, Lutterworth, UK) at a density of 5 × 10^4^ cells/ml. 24 hours later the cells were fixed with 4% PFA for 10 min at RT. After one hour blocking with 10% normal goat serum (Sigma Dorset, UK,), the cells were incubated with antibodies against ITCH (BD, Wokingham, UK, 1:100), p73 (Generon, Slough, UK, 1:500), integrin αv (R&D, Cleckheaton, UK, 1:100), β3 (Generon, Slough, UK, 1:100) and β5 (R&D, 1:100) at 4 °C overnight, followed by one hour incubation with Alexa-488 or Alexa-594 conjugated corresponding secondary antibodies (1:500, Thermo Fisher, Paisley, UK). The coverslips were then mounted with hydromount (Sigma, Dorset, UK) mounting medium containing 10 µg/ml DAPI and images were acquired using the Metamorph software.

### Western blot

72 hours after transfection, protein was extracted in RIPA (Radio-Immunoprecipitation Assay) lysis buffer (Sigma, Dorset, UK) containing a complete protease inhibitor cocktail (1:100, Roche, Welwyn Garden City, UK). 100 µl of lysis buffer was added to each well of the 6-well plate, and left on ice for 10 minutes. Samples were collected and boiled for 3 min and then centrifuged at 14,000 × *g* for 10 min at 4 °C. The supernatants were stored at −80 °C until needed. 30 µl /well of each sample were loaded onto NuPAGE Novex 10% bis-Tris Gel, and run at a constant voltage of 150 V for 1 hour, before being transferred to a nitrocellulose membrane at 300 mA for 2 hours. The membrane was then blocked with Odyssey block solution (LI-COR Biosciences, Cambridge, UK) for 60 min, incubated by primary antibodies of mouse anti-ITCH antibody (1:2000, BD, Wokingham, UK) or rabbit anti-TP73 (1:1000, Generon, Slough, UK), and mouse anti-beta actin (1:5000, Sigma, Dorset, UK) overnight at 4 ºC. After washing with PBS containing 1% Tween 20 (PBST) for 15 min × 3 times at room temperature, the membrane was then incubated with IRDye 680 RD goat anti-rabbit or IRDye 800CW goat anti-mouse 2^ry^ antibodies (1:15000, LI-COR Biosciences, Cambridge, UK) for 1 hour at RT. The image of the blotted membrane was acquired by the Odyssey Clx infrared imaging system (LI-COR Biosciences, Cambridge, UK) using image studio software. For quantification of the ITCH or TP73 expression, the intensity of the ITCH or TP73 protein bands was measured using Image Studio Lite ver 5.2, and normalized against the intensity of the corresponding beta-actin band.

For western blotting of integrins, the primary antibodies used were: goat anti-human integrin alpha V (1:1000, R&D, Cleckheaton, UK) or mouse anti-human integrin beta 3 (1:1000, R&D, Cleckheaton, UK) or Sheep anti-human integrin beta 5 (1:1000, R&D, Cleckheaton, UK) followed by the corresponding anti-goat/sheep/mouse IRDye 680 RD donkey anti-goat/sheep and IRDye 800CW goat anti-mouse 2^ry^ antibodies (1:15000, LI-COR Biosciences, Cambridge, UK) for 1 hour at RT. The membrane was then visualized using the Odyssey Clx infrared imaging system.

### SubG1 analysis

To examine the effects of ITCH silencing on the apoptosis of neuroblastoma cells upon irradiation, Kelly cells were first transfected with ITCH siRNA as described above, using irrelevant siRNA as a control. Cells were exposed to 4 Gy irradiation three days after transfection then 24 hours after irradiation, cells were trypsinized and centrifuged at 500 x *g*, for 5 min. Pre-cold 70% ethanol was then added dropwise to the cell pellet, and the cells were fixed at 4 °C for 30 min before repeated centrifugation and the cell pellet was washed with citrate buffer (0.2 M Na_2_HPO_4_ and 0.1 M Citric acid) twice before adding 50 µl RNaseA (100 µg/ml) followed by 50 µg/ml propidium iodide (PI) for 5 min. The cells were then pelleted again at 500 x *g* for 5 min, and then re-suspended in 0.5 ml PBS for FACS analysis. The cell cycle profile was acquired using a FACS calibur machine (BD, Wokingham, UK) and the percentage of cells in the SubG1 region was calculated using the Flowjo software (Flowjo, LLC).

### Establishment of the xenograft neuroblastoma model and *in vivo* transfection of ITCH siRNA with nanoparticles

6–8 week old NOD-SCID gamma (NSG) female mice were ordered from Charles River laboratories, Harlow, UK. The mice were acclimatized for 1–2 weeks before engrafting the human neuroblastoma cells. On the day of engraftment, Kelly cells were harvested by trypsinisation and re-suspended in RPMI 1640 medium at a density of 3 × 10^6^ cells/100 µl. 100 µl cell suspension was then mixed with an equal volume of Matrigel (BD biosciences) on ice and immediately injected subcutaneously into the left or right upper flank of the mouse. The body weight and the tumour size/volume of each mouse were monitored from 4 days after engraftment onwards. The *in vivo* administration of the ITCH siRNA nanoparticles was performed on mouse once its tumour grew bigger than 5 mm at both dimensions. The nanoparticle formulation for the *in vivo* study was also prepared at the 1:4:1 weight ratio, using cationic lipid AT-1, which contains 1% PEG, and the peptide used was ME27. 1 mg siRNA/kg was injected intravenously into each mouse. The tumours were harvested either 24 hours or 48 hours after administration, and processed for qPCR analysis.

### Real time RT-PCR analysis of tumour samples

Each tumour was divided into 4 parts and RNA was extracted respectively for real time RT-PCR analysis. iTaq Universal SYBR Green One-Step Kit (Bio-rad, Watford, UK) was used in this assay. For normalization, 2 separate housekeeping genes, beta-actin and SDHA^37^ were included. As we had in total 104 samples to test, it was not possible to fit all samples in a single 96-well testing plate. Therefore, we included one sample from an untreated tumour as a common control. This sample was incorporated in every plate we ran and all the data from each plate were calculated relative to this sample, in other words, the ddCT value of each sample was calculated by subtracting the dCT value of this common control sample.

## Supplementary information

supplementary information.
