# Supplementary material for: Silencing E3 Ubiqutin ligase ITCH as a potential therapy to enhance chemotherapy efficacy in p53 mutant neuroblastoma cells
Source: Sci Rep. 2020 Jan 23;10:1046. doi: 10.1038/s41598-020-57854-6 (PMC6978385; doi:10.1038/s41598-020-57854-6)
Supplement: Supplementary file 1 — supplementary information. [file 41598_2020_57854_MOESM1_ESM.pdf]

Title:

Silencing E3 Ubiqutin ligase ITCH as a potential therapy to enhance chemotherapy efficacy in p53 mutant neuroblastoma cells

Running title:

In vitro and *in vivo* silencing of ITCH in neuroblastoma models

Jinhong Meng[1,2], Aristides D Tagalakis[2]*, Stephen L Hart[2]**.

1. Ryboquin Ltd, Ettrick Riverside, Dunsdale Road, Selkirk, TD7 5EB, UK.
2. Genetics and Genomic Medicine Programme, UCL Great Ormond Street Institute of Child Health, 30 Guilford Street, London, WC1N 1EH, UK.

* Current address: Department of Biology, Edge Hill University, Ormskirk, L39 4QP, UK

**To whom correspondence should be addressed:

S. L.H (s.hart@ucl.ac.uk)

UCL Great Ormond Street Institute of Child Health, 30 Guilford Street, London, WC1N 1EH, UK.

Telephone: 0044-2079052228

Email: S.hart@ucl.ac.uk

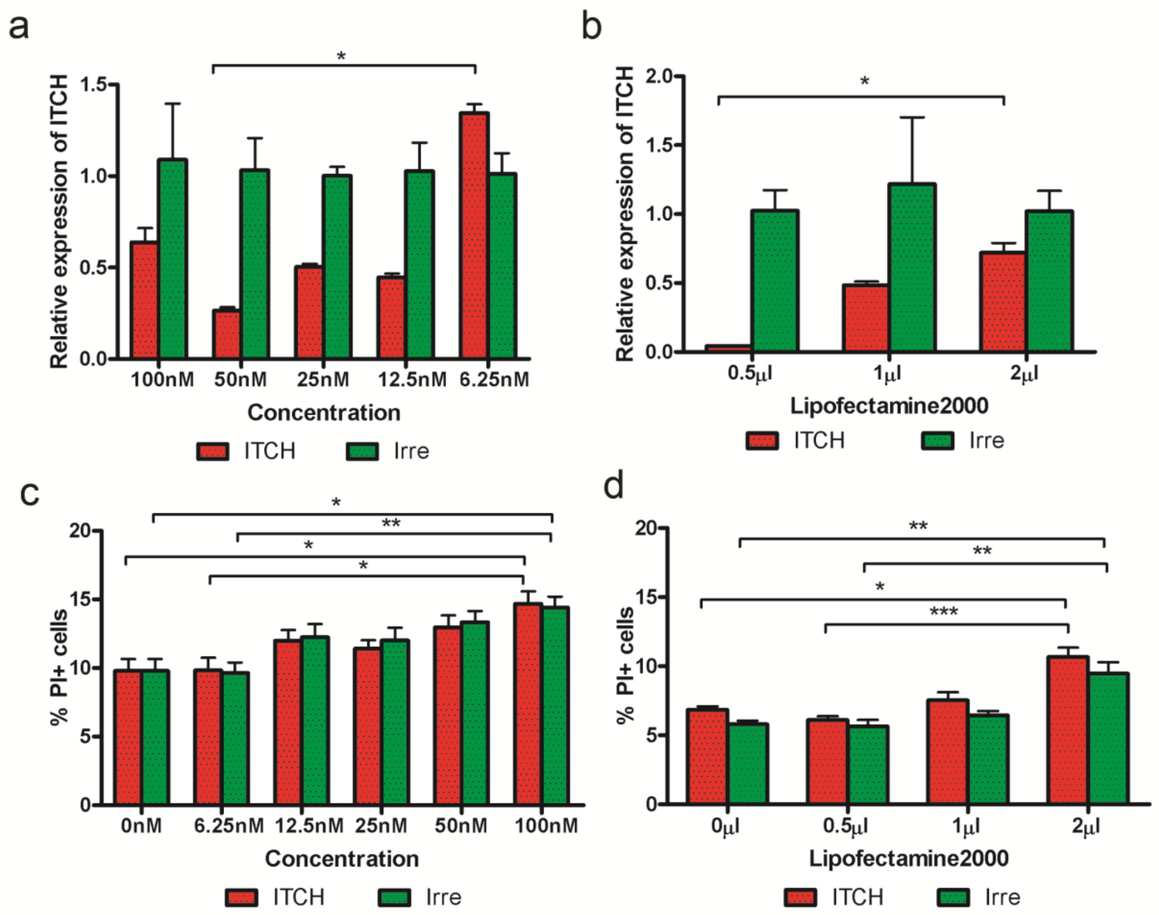

Supplementary Figure 1. Knockdown of ITCH *in vitro* on BE2 cells using different concentration of ITCH siRNA (a, c); or different amount of L2K reagent (b, d). The cell viability in each transfection condition is indicated by %PI+ cells after transfection (c and d).

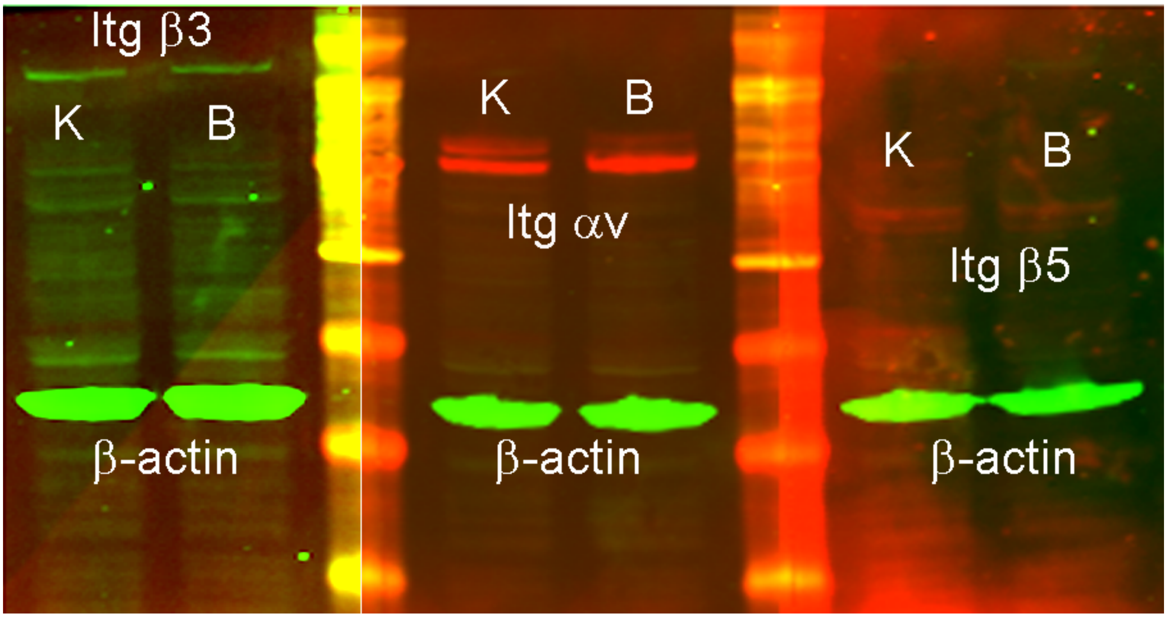

Supplemenary Figure 2. Full-length western gel of Figure 2b. The same protein samples (Kelly and BE2) were loaded on the same gel for 3 times. After separating and transferring, the membrane was cut to 3 parts and blotted with antibodies against integrin beta3, integrin beta 5 and integrin alpha v, followed by corresponding 2nd antibodies. The western blot was visualized using odyssey fluorescent system.

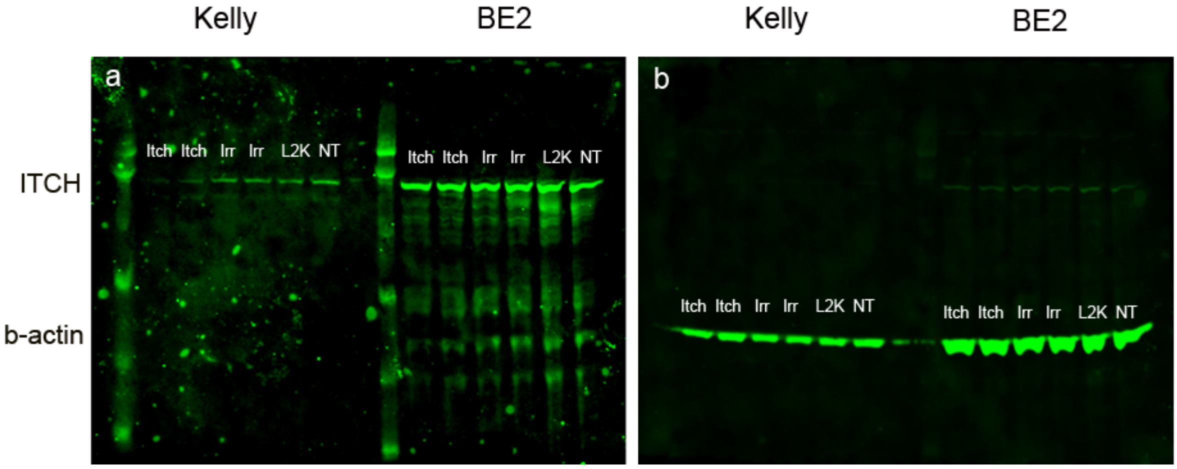

Supplementary Figure 3. Full-length western blot of Figure 3B. The protein samples of Kelly cells or BE2 cells treated the same way by Itch SiRNA or irrelative SiRNA were blotted firstly by ITCH antibody (a), to show the expression level of ITCH after the treatment; then the same membrane was blotted by beta-actin (b), to show the loading control expression.

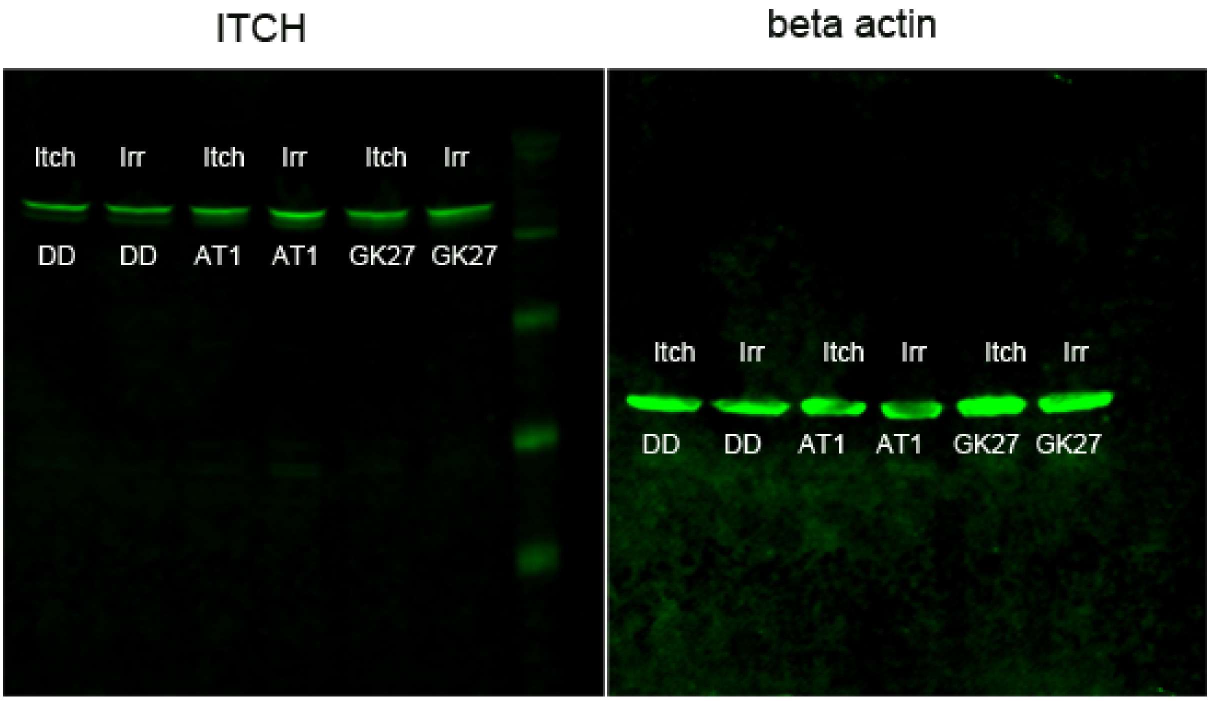

Supplementary Figure 4: Full-length western blot of Figure 3C-c. The protein samples of Kelly cells transfected by Itch SiRNA or irrelative SiRNA using nanoparticles (DD, AT1 and GK27) were blotted by ITCH antibody (left panel), to show the expression level of ITCH after the treatment; then the same membrane was blotted by beta-actin (right panel), to show the loading control expression.

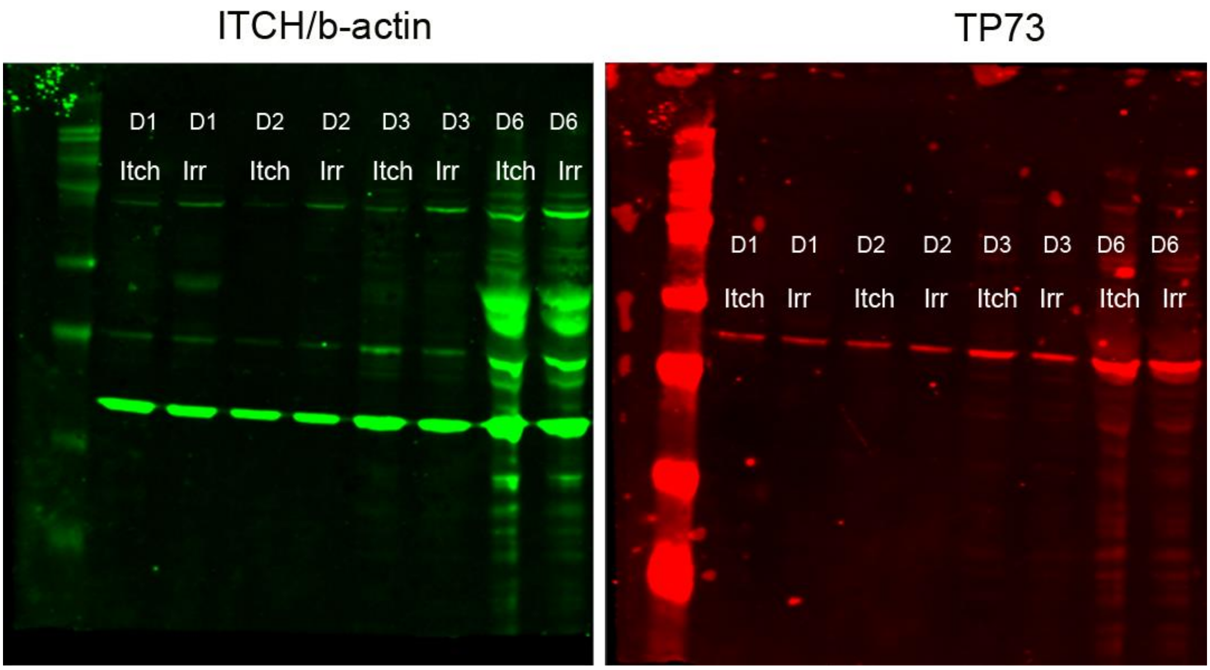

Supplementary Figure 5: full length western blot of Figure 4a showing silence of ITCH protein and upregulation of TP73 after transfection of ITCH siRNA in Kelly cells. Image in right panel shows the blotting result of ITCH and beta-actin, (green channel, 800nm), while the image in left panel shows the same membrane blotted by TP73 antibody (red channel, 680nm).
